# Evaluation of the Accuracy of Panoramic Radiography in Linear Measurements of the Jaws

**Published:** 2011-09-25

**Authors:** S. H. Hoseini Zarch, A. Bagherpour, A. Javadian Langaroodi, A. Ahmadian Yazdi, A. Safaei

**Affiliations:** 1 Assistant Professor, Department of Oral and Maxillofacial Radiology, School of Dentistry and Dental Research Center, Mashhad University of Medical Sciences, Mashhad, Iran; 2 Assistant Professor, Department of Oral and Maxillofacial Radiology, School of Dentistry, Hamedan University of Medical Sciences, Hamedan, Iran; 3 Consultant Professor, Department of Oral and Maxillofacial Radiology, School of Dentistry and Dental Research Center, Mashhad University of Medical Sciences, Mashhad, Iran; 4 Private Dental Practice, Mashhad, Iran

**Keywords:** Panoramic Radiography, Linear Measurement, Mandibular Canal, Mental Foramen

## Abstract

**Background/Objective:**

Panoramic radiography has a great place among imaging techniques because of its enormous advantages. One of the characteristics of an ideal imaging technique is to supply precise measurement. The purpose of the current study was to evaluate the accuracy of linear measurements of the jaws on panoramic radiographs.

**Patients and Methods:**

In this study, the vertical distances between the metal markers were measured by panoramic radiography in seven sites of two skulls in various head positions. Then the radiographic measurements were compared with the actual values.

**Results:**

Eighty three percent of the measurements were underestimated, 8.5% were overestimated on panoramic radiography and 8.5% of the measurements had no difference with the real measurements. Overestimation was not greater than 1 mm. The difference between actual and radiographic measurements was less in the posterior areas and in the mandible . In all head positions, the greatest difference between actual and radiographic measurements occurred in the anterior area.

**Conclusion:**

Based on the results of this study, linear measurements on panoramic radiography are more reliable in the posterior areas and may be used in early clinical measurements.

## Introduction

To select a radiography method in addition to clinical investigations and patient’s history, one should consider the diagnostic quality of the image, region of interest, radiation dosage and accessibility.[1]

Panoramic radiography is one of the most common extraoral techniques which provides a precise view of the maxillomandibular area presenting a unique image of both upper and lower dental arches.[2][3] This imaging method provides a better view of the bone structure, especially the lower jaw (mandible) and could be a good guide for the examination prior to place an implant, showing the relation between the location of the surgery and the adjacent anatomical structures such as the mandibular canal and the mental foramen.[1][4] Other advantages of this technique include a lower radiation intake for the patient and a relatively shorter imaging time.[2][3]

However, panoramic radiography similar to other methods has its own limitations such as lower image clarity compared with periapical radiography, high magnification and distortion, low image resolution, and 2 dimensional images without any sectional information.[1][2][3] Because of a relatively thin focal trough layer especially in the anterior locations, this technique is very sensitive to head positions. Focal trough is 4.5-12 mm on the anterior area and 2 to 3 times bigger on the posterior region.[5][6] Mistakes due to the patient’s position could increase errors of horizontal dimension measurements questioning the accuracy of this technique in delicate situations such as placing an implant.[7][8]

In order to justify the ambiguities and to perform better investigations, this study is conducted to evaluate the accuracy of panoramic radiography in vertical dimension measurements of the jaws with different head positions.

## Patients and Methods

According to similar studies and due to ethical considerations, the study was performed on two dry human skulls. In each skull, seven locations on the alveolar crest region, which includes almost all dental locations in both jaws, were marked for the linear measurements. The locations include central dental areas (21) and the second molar (27) on the left side and second premolar (15) on the right side and of the upper jaw and central, (31) canine, (33) second premolar (35) and first molar (36) on the left side of the lower jaw. These locations are marked using 3mm diameter lead balls. The lead balls were placed on the crest edge in different distances on the buccal and lingual surfaces, midline and inferior border.

**Fig. 1:**
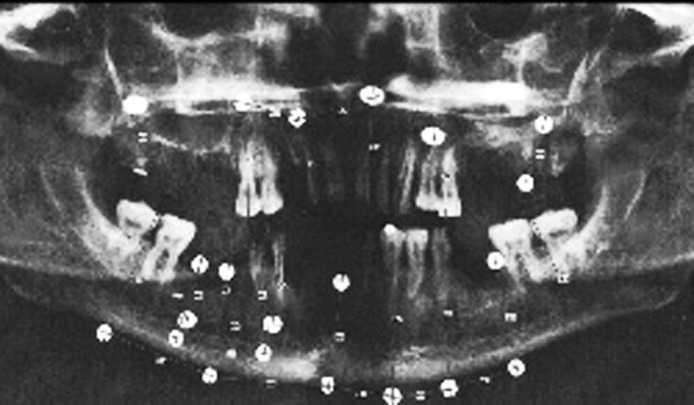
Skull number 1 on normal head position. Panoramic radiograph shows lead markers in different regions of the jaws

To obtain panoramic images, Planmeca Promax digital panoramic (Planmeca Co, Helsinki, Finland) with 2.5mm total aluminum filtration and 60kvp and 4mA kilo voltage adjustment is utilized. The images were saved in jpg format (Fig. 1). Skull position adjustment was carried out regarding light rays relative to sagittal, Frankfort lines and lip line relative to the focal trough. Finally, panoramic images were taken in five different incorrect head positions which more commonly occurred and a normal head position by the oral and maxillofacial radiologist. Incorrect positions were as follows:

1. The chin was tipped downward (15°).

2. The chin was tipped upward (15°).

3. The head was tilted toward film (10°).

4. The head was positioned backward relative to the focal trough (5mm).

5. The head was positioned forward relative to the focal trough (5mm).

The vertical height was defined as the distance between the lateral sides of the two lead balls one of which was placed on the crest edge and the other on different locations of the buccal and lingual surfaces or the inferior border of the mandible in each panoramic image. This was calculated by Planmeca Romexis 2.2.4R software. In addition, the results were remeasured using regular caliper with 0.1mm accuracy on the real skulls and registered. Microsoft Office Excel 2007 was used for drawing Bland-Altman plots and tables.

## Results

**Fig. 2:**
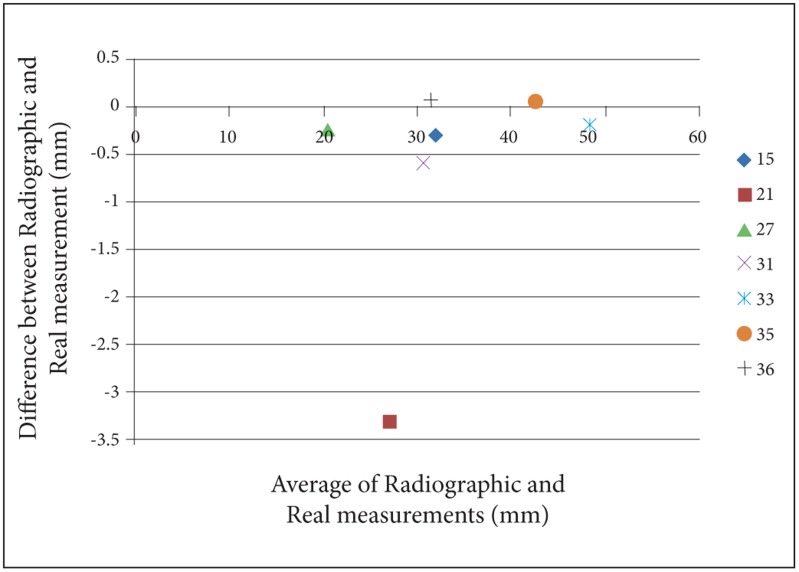
Bland-Altman plot for normal head position.

**Fig. 3:**
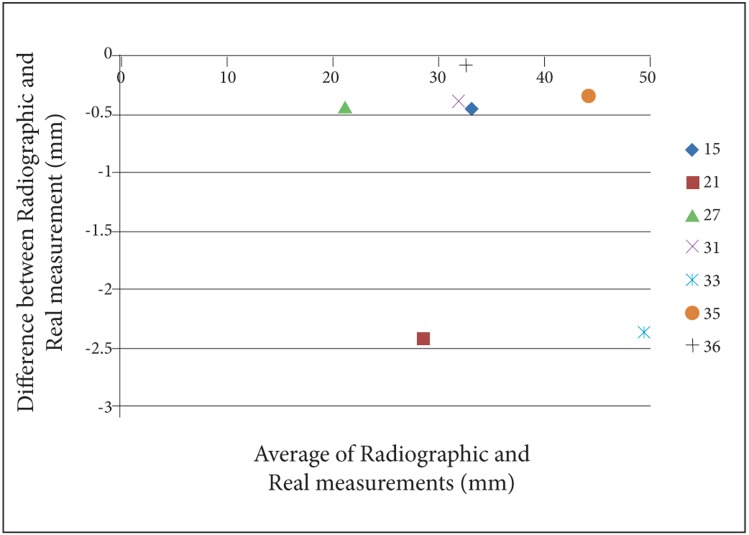
Bland-Altman plot for the chin tipped downward (15°).

**Fig. 4:**
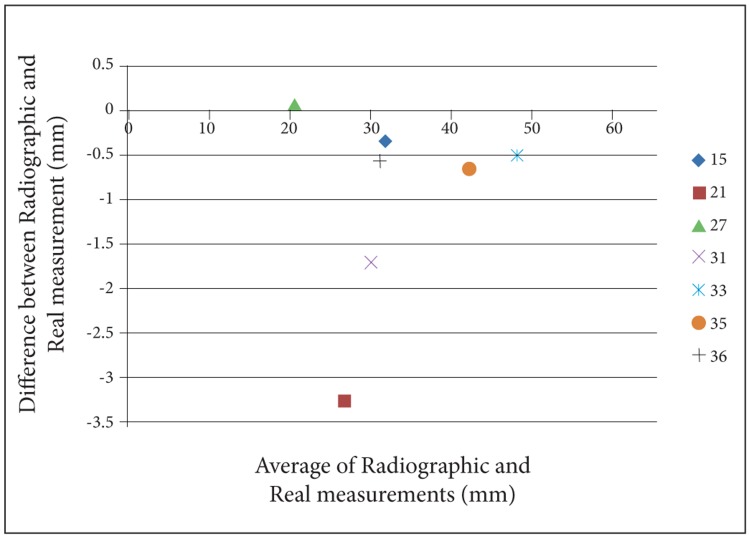
Bland-Altman plot for the chin tipped upward (15°).

**Fig. 5:**
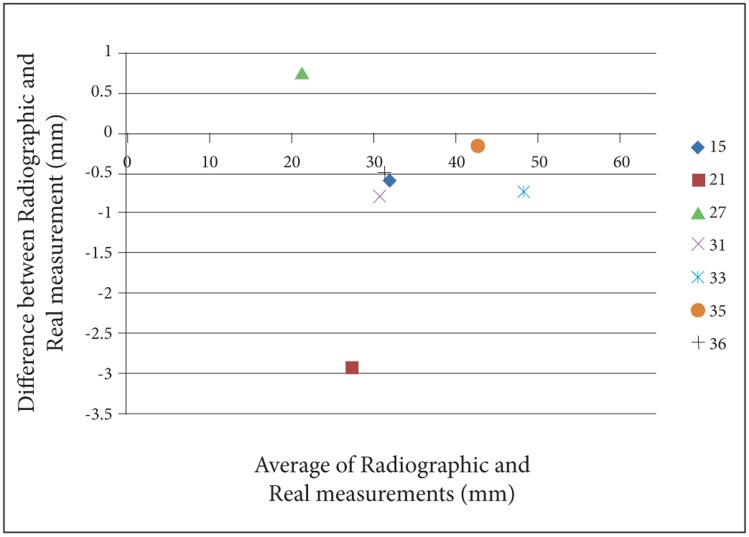
Bland-Altman plot for the head tilted toward film (10°).

**Fig. 6:**
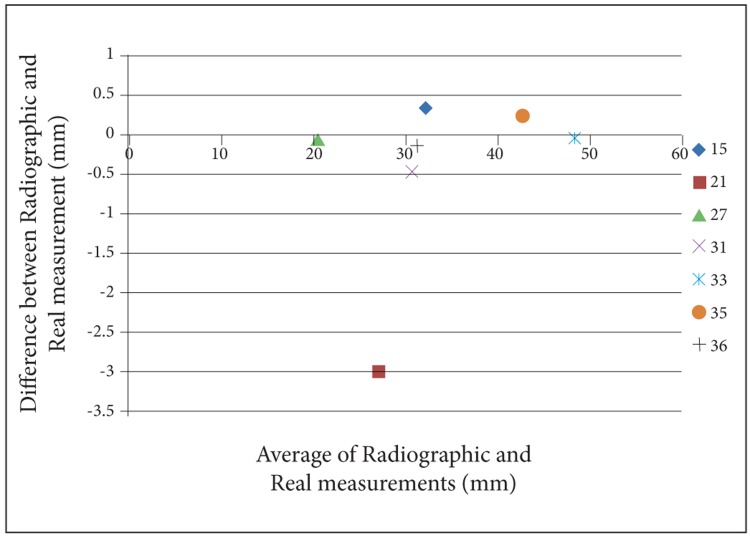
Bland-Altman plot for the head positioned backward relative to the focal trough (5mm).

**Fig. 7:**
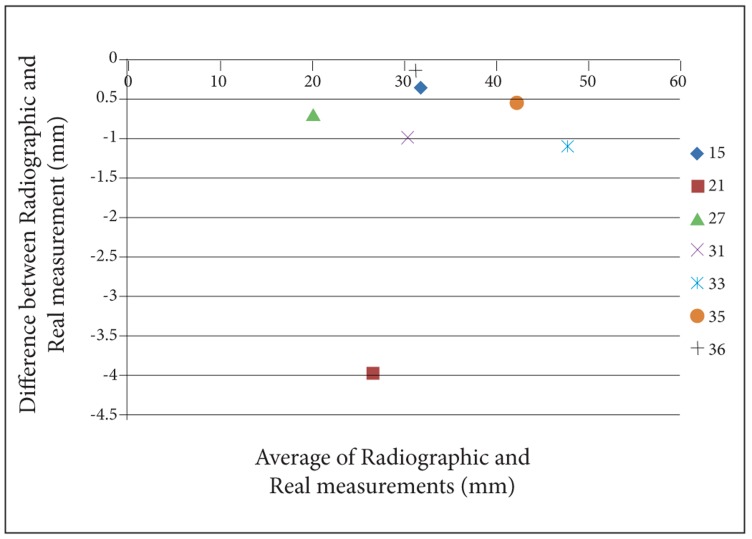
Bland-Altman plot for the head positioned forward relative to the focal trough (5mm).

For a better perception of the results, we used Bland- Altman plots to compare magnification percentages of seven different dental locations on six different head positions. To prevent complexity of Bland-Altman plots, all the measurements on the two skulls were averaged. (Figs. 2, 3, 4, 5, 6 & 7)

On the normal head position, the difference between panoramic radiography measurements and real measurements was calculated from 0 to 3.3mm. The highest difference among the measurements on a normal head position was on location 21 and the lowest was on locations 35 and 36. Besides, these locations were the only places where the normal head position measurements estimated higher than the real values (magnification rate was 0.2). The lowest underestimation rate on a normal head position was for location 33 with the amount of 0.1.

When the chin tipped downward (15°), except for location 36 on skull number 1 (the difference was 0), the results of all locations were underestimated. The highest difference was for locations 21 and 33 with the amount of 2.4 and the lowest was for location 36 with the amount of 0.2.

On the chin tipping upward (15°), except for location 27, the results were underestimated. About location 27 on skull number 2, the difference was 0 and on skull number 1, it had 0.5% overestimation rate. The highest underestimation rate was for location 21, and the lowest was for location 15.

On the head tilting toward film (10°), for location 35 on skull number 1, the difference was 0. However, other locations were underestimated with the highest rate for location 21 and the lowest for locations 35 and 36.

When the head was positioned backward to the focal trough (5mm), only locations 15 and 35 showed overestimation, while the highest underestimation rate was for location 21 and the lowest was for locations 27, 33 and 36. On the head position forward to the focal trough (5mm), all locations were underestimated with the highest rate for location 21 and the lowest for location 36.

In general, the results were underestimated in 83% of the measurements, 8.5% of the panoramic image measurements had no difference with the real head measurements and 8.5% were overestimated. Twentyfour percent of the measurements were more than 1mm underestimated and overestimation was not greater than 1mm. (Table 1 & 2)

**Table 1:** The Difference Between Panoramic Radiography and Real Measurements on Skull Number 1 (mm) in Six Different Head Positions

**Region**	**Dental Locations**	Head Position *					
		**1**	**2**	**3**	**4**	**5**	**6**
Maxilla	21	-3.3	-2.4	-3.2	-4.6	-3	-4
	15	-0.2	-0.4	-0.4	-0.7	+0.3	-0.3
	27	-0.3	-0.5	+0.1	-0.4	0	-0.7
Mandible	31	-0.6	-0.3	-1.8	-1.4	-0.4	-1.4
	33	-0.1	-2.3	-0.5	-0.3	0	-0.9
	35	+0.1	-0.4	-0.7	0	+0.2	-0.6
	36	+0.1	0	-0.7	-0.1	-0.2	-0.1

^*^ 1: Normal Head Position; 2: The chin was tipped downward (15°); 3: The chin was tipped upward (15°); 4: The head was tilted toward film (10°); 5: The head was positioned backward relative to the focal trough (5mm); 6: The head was positioned forward relative to the focal trough (5mm).

**Table 2:** The Difference Between Panoramic Radiography and Real Measurements on Skull Number 2 (mm), in Six Different Head Positions

**Region**	**Dental Locations**	Head Position *					
		**1**	**2**	**3**	**4**	**5**	**6**
Maxilla	21	-3.3	-2.4	-3.3	-4.5	-3	-4
	15	-0.4	-0.5	-0.3	-0.6	+0.6	-0.4
	27	-0.2	-0.4	0	-0.3	-0.1	-0.7
Mandible	31	-0.6	-0.5	-1.6	-1.4	-0.5	-0.6
	33	-0.3	-2.4	-0.5	-0.4	-0.1	-1.3
	35	0	-0.3	-0.6	-0.1	+0.3	-0.5
	36	0	-0.2	-0.4	-0.1	-0.1	-0.2

^*^ 1: Normal Head Position; 2: The chin was tipped downward (15°); 3: The chin was tipped upward (15°); 4: The head was tilted toward film (10°); 5: The head was positioned backward relative to the focal trough (5mm);6: The head was positioned forward relative to the focal trough (5mm)

## Discussion

Panoramic radiography is a common imaging technique in dentistry that provides a unique image of both upper and lower dental arches. Relatively low radiation and time and budget saving are other advantages of this imaging technique.[9][10] Despite such advantages, magnification, high distortion and possible mistakes due to incorrect head adjustment are the main disadvantages.[7][11][12] Because of a rather thin focal trough or image layer especially on the anterior region, this imaging method is sensitive to different head positions.[5][6] In addition, parameters such as imaging device, equipment and the patient’s position could affect the panoramic image quality and consequently the clinical judgement. In this study, we used digital panoramic radiography, which increases the image quality and reduces the patient's radiation intake.[13]

Measurements were performed for six different head positions and the effects of these different positions on the accuracy of measurements were surveyed.

Sonic[14] compared the accuracy of periapical radiography, panoramic radiography and CT scan in localizing the mandible canal on a dry human mandible. The difference between panoramic radiography measurements and real measurements on normal head position was calculated from 0.5 to 7.5 mm (mean, 3 mm). However, in our study this calculation ranges from 0 to 3.3 mm. They also reported most of the results as overestimated, which was the opposite of our findings. In the present study, results were underestimated in 83% of the measurements.

In another study, Peker [3] surveyed three different imaging techniques, including panoramic radiography, conventional tomography and CT scan to localize mandible canal location before placing an implant. To measure vertical distances for different posterior locations, six dry human mandibles were used. There was no significant difference between real measurements and panoramic measurements. There were no overestimations more than 1mm which were the same as our study. Besides, in Peker’s study, 20% of the measurements were more than 1mm underestimated. However, in our study this rate was 24%. This difference could be due to the different locations and also different head adjustment positions in our study.

Lucchessi [15] showed that deviation from real measurements for the anterior mandibular locations compared with the other locations is more common in panoramic radiography. In our study, the highest differences obtained for all skull positions were in the anterior locations and increased by approaching the midline.

Bou Serhal [16] evaluated the accuracy of panoramic radiography, CT scan and spiral tomography in localization of the mental foramen. The results revealed that the majority of the measurements in panoramic radiography overestimated the real measurements. Another study by Akdeniz et al.,[17] which was aimed on the evaluation of height and bone density in panoramic radiography and regular tomography showed the overestimated measurements for the panoramic technique.

Rockenbach [18] conducted a study with the purpose of evaluating mandible implant location with panoramic radiography and conventional tomography on 20 dry human hemimandibles. The results showed overestimated measurements compared with real measurements for both techniques. The results of the three previous studies were the opposite of our findings.

Reddy [19] compared advantages of panoramic radiography and conventional tomography prior to placing an implant and concluded that utilizing panoramic radiography alone results in underestimation of the real size of the implants. In addition, Lindh [20] concluded that panoramic radiography underestimates the real distance from the crest to the upper canal border. The results of the two previous studies were in acceptance with our results.

In conclusion, results of the present study showed that in the majority of locations (83%) panoramic measurements were underestimated. The highest differences were for anterior locations on all head positions. However, all the measurements of the anterior locations were underestimated and radiographic measurements for the posterior locations, especially in the mandible were more reliable. It is possible to achieve approximately precise measurements from panoramic radiographs for posterior regions of the jaws, since the patient’s head position is adjusted correctly. So the panoramic radiographs besides providing a broader visualization of the jaws and adjoining anatomic structures, may be used as an early assessment instrument for implant planning.
